# Predictive Models for Equine Emergency Exploratory Laparotomy in Spain: Pre-, Intra-, and Post-Operative-Mortality-Associated Factors

**DOI:** 10.3390/ani12111375

**Published:** 2022-05-27

**Authors:** Manuel Iglesias-García, Isabel Rodríguez Hurtado, Gustavo Ortiz-Díez, Jorge De la Calle del Barrio, Cristina Fernández Pérez, Raquel Gómez Lucas

**Affiliations:** 1 Department of Equine Surgery, University of Extremadura, 10004 Caceres, Spain; 2 Large Animal Department, Alfonso X el Sabio University, 28691 Madrid, Spain; irodrhur@myuax.com (I.R.H.); jdelbdel@uax.es (J.D.l.C.d.B.); rgomeluc@uax.es (R.G.L.); 3 Department of Animal Medicine and Surgery, Complutense University of Madrid, 28040 Madrid, Spain; gusortiz@ucm.es; 4 Servicio de Medicina Preventiva, Área de Santiago y Barbanza, Instituto de Investigaciones Sanitarias de Santiago (IDIS), 15706 Santiago de Compostela, Spain; cristina.fernandez.perez3@sergas.es

**Keywords:** epidemiology, horse, colic, survival, predictive

## Abstract

**Simple Summary:**

Colic syndrome poses a potentially devastating situation in equine patients. Therefore, the prediction of survival has been an important research area over the years, especially in surgical cases. However, prognoses and survival rates are highly diverse in different populations. Many clinically relevant and numerical variables have been used in several large-scale studies. We aimed to determine the survival percentage and other predictive variables in a specific population with concrete characteristics in Spain, in an attempt to predict prognosis and survival using only easily accessible variables.

**Abstract:**

The extrinsic and intrinsic characteristics of an equine population may influence the onset of gastrointestinal lesions and affect the survival rate of patients. The equine population in Spain has been the focus of a small number of studies, none of which have involved more than one surgical center. In this retrospective cohort study, we aimed to analyze the survival rate, identify the variables that influenced death, and generate multivariate models using clinical variables. Data were collected from the clinical records of two surgical referral centers in the same region, and a total of 566 horses met the inclusion criteria. The statistical analysis was divided into three parts: The first and second included logistic analysis, in order to identify the variables most closely associated with survival. The third part assessed all previous variables in terms of survival and hospitalization time, using a COX survival analysis. The main risk factors associated with intra-operative mortality were related to seasonality (winter and summer), patient age (older than 9 years), distance from the hospital, the presence of a strangulating lesion, and the bowel segment affected (small intestine). Furthermore, the main factors associated with mortality during hospitalization were the characteristics of the lesions (strangulating) and the differences between surgical centers. The models generated in this study have good predictive value and use only reliable and easily obtainable variables. The most reliable characteristics are those related to the type of colic and the location of the lesion.

## 1. Introduction

Abdominal acute syndrome, or colic, is a very important disease among horses, the incidence of which is difficult to assess precisely. It has been suggested that approximately 1–17% of horses with colic require surgical treatment [1,2,3,4,5,6]. Some progress has been made in the last few years towards increasing survival rates, based on changes in surgical techniques [2]; however, horses submitted to colic surgery still have an increased risk of death compared to other surgical procedures [7,8]. Therefore, identifying the associated risk factors is an important issue when evaluating colic survival rates. 

There have been many retrospective studies focusing on survival rates and risk factors in thoroughbred populations [9,10,11] in the U.K. and USA [1,12,13]. It is well-known that each population has its own specific characteristics in terms of training, management, diet, weather conditions, and so on, all of which are important in the development of colic. As a result, many articles highlighting the specific conditions in different countries have been published [14,15,16], which is crucial for the development of comparative studies that allow us to identify and differentiate between the most important risk factors. 

In Spain, no studies have been conducted involving more than one hospital, and only two articles have evaluated the survival rates and risk factors in the country’s equine population. One of these studies was conducted in the north of Spain [17], and the other was conducted in a single surgical facility in the center of Spain [18]. Our objectives were to analyze the risk factors in horses surgically treated for colic in Spain and to create predictive models using only very simple variables.

## 2. Materials and Methods

This is a multicentric retrospective cohort study. The clinical records of all colic surgeries performed in two referral centers were revised for analysis. All the patients’ owners provided consent for the surgery, further treatments, and use of the information for research. Both referral centers are situated in the Madrid region of Spain. Records from 2006 to 2015 were reviewed, and all of the horses that had been submitted to an emergency exploratory laparotomy were considered for the study. A total of 636 exploratory laparotomies were reviewed, 566 of which (88.9%) met the inclusion criteria and were therefore included in the study. We excluded horses that had been operated on more than once, data from second surgeries, incomplete records, horses younger than 1 year of age, and horses that were submitted to an exploratory laparotomy due to non-gastrointestinal causes. The study population comprised 41.6% stallions, 36.4% geldings, and 22.0% mares, with a median age of 9 years (interquartile range ,IQR 7–13). The most frequent breed was Spanish Pure Breed, comprising 39.9% of the population.

The variables assessed were patient age, sex, and breed; date of surgery (grouped into months); environmental temperature (at time and place where the pathology began), and geographic origin (the distance between the place where the horse was housed at the onset of colic and the referral center, measured in kilometers). Regarding the date of surgery variable, the patients were also grouped according to month and season. All weather-related data were obtained from the State Meteorological Agency of Spain. 

The clinical data variables collected were as follows: lesion (specific type of lesion); anatomical localization of the lesion (small or large intestine); classification according to vascular involvement (strangulating or non-strangulating lesion); surgical procedure performed (exploratory laparotomy (EL), exploratory laparotomy with enterectomy and anastomosis (ELA), or exploratory laparotomy with enterotomy (ELE)); number of days of hospitalization required; and final outcome. Regarding the final outcome, the patients were divided into the following three categories: died or euthanized during the surgical procedure, alive during the surgery but died during hospitalization, or discharged. 

### Data Analysis

The dependent variable for the pre- and intra-operative study was death. In the post-operative study, the dependent variables were death and median survival time.

Categorical variables were presented as percentages. For continuous variables, data distribution normality was evaluated through the Kolmogorov–Smirnov test. The results of the normality test were statistically significant (*p* < 0.001) and were provided as median and IQR. Continuous data were presented as mean (±standard deviation) or median (interquartile range, IQR).

For the data treatment, the temperature considered for analysis was the monthly average temperature where the horse was housed when the problem appeared. The environmental temperature was divided into quintiles and separated by cut-off points for subsequent statistical evaluation. A cut-off point was also set to define whether or not the horse was in the same area as the surgical center at the moment of onset.

Multivariable logistic regression was used to detect factors associated with death in two scenarios, the pre-operative and the intra-operative model; the results are presented as the odds ratio (OR). To calibrate the model, we performed a Hosmer–Lemeshow test. To discriminate the model, we used the ROC (Receiver Operating Characteristic) curves. 

To evaluate the hospitalization risk factors, we created a multivariable COX regression model. The final model was built by means of stepwise forward selection and backward elimination. The significance levels for selection and elimination were *p* < 0.05 and *p* ≥ 0.10, respectively. We used Harrel’s C test to discriminate the model. Bootstrapping was used to assess the internal validity of the model.

We used the Stata statistical package (13.1 StataCorp., College Station, TX, USA) and SPSS statistical software (IBM, Armonk, NY, USA) for analysis. All *p*-values were two-sided, and *p* < 0.050 was considered statistically significant.

## 3. Results

### 3.1. Seasonality and Geographic Origin 

August was the month with the highest percentage of surgeries (12.7%). When grouping the months into seasons, summer was the season with most cases (33.4%). Most patients came from the Madrid region, where the referral hospitals were located (70.3%). Distance to the surgical center had similar results, and a cut-off point of 70 km was found to be significant. 

### 3.2. Intestinal Lesions

The most frequently diagnosed lesion was a colon torsion, with 89 cases (16.1%). When grouping the lesions according to location, the large intestine was the most frequent, with 52% of the cases. Strangulating lesions were the most frequent type of lesion, with 54.6% of the cases. The most common procedure performed was laparotomy and manipulation alone, which was carried out in 57.5% of the horses. The median hospitalization time was 9.67 days (IQR, 3–12). Finally, 63.6% of the horses were alive at discharge, 22.3% died during surgery, and 14.1% died during hospitalization. 

### 3.3. Factors Associated with General/Pre-Operative Variables

We used multivariable logistic regression to detect the factors associated with death in the pre-operative model by considering variables related to the patient but not to the pathology. 

For the pre-operative period, the variables selected in the predictive model were as follows (Table 1): season (winter and summer; OR = 1.8; 95% Confidence Interval (CI): 1.2–2.8, *p* = 0.006); patients older than 9 years (OR = 2.0; 95% CI: 1.3–3.1, *p* = 0.001); and distance over 70 km from the referral hospital (OR = 1.7; 95% CI: 1.1–2.7, *p* = 0.023). The Hosmer–Lemeshow test resulted in a *p*-value of 0.955 and an Area Under the Curve (AUC) of 0.635 (95% CI: 0.578–0.692).

### 3.4. Factors Associated with Surgery-Related Variables

The second multivariable logistic regression model included intra-operative variables, such as the section of the intestine affected and the type of lesion, in addition to the pre-operative factors previously described. When analyzing the variables obtained in the intra-operative period, the significant variables selected were (Table 2): season (winter and summer; OR = 1.8; 95% CI: 1.1–2.9, *p* = 0.017); patients older than 9 years (OR = 1.8; 95% CI: 1.1–2.9, *p* = 0.016); classification of the lesion as strangulating (OR = 5.3; 95% CI: 2.7–10.0, *p* < 0.001); and, finally, the location of the lesion in the small intestine (OR = 1.7; 95% CI: 1.0–3.0, *p* = 0.041). The Hosmer–Lemeshow test resulted in a *p*-value of 0.901 and an AUC of 0.756 (95% CI: 0.707–0.805). 

### 3.5. Factors Associated with Survival during Hospitalization

The final variables identified by the model as most strongly associated with death were: the strangulating characteristics of the lesion (Hazard Ratio (HR) = 1.5; 95% CI: 0.1–2.4, *p* = 0.004) and the difference between the surgical centers in which the horses were operated on (HR = 2.6; 95% CI: 1.6–4.1, *p* < 0.001). Harrell’s C test resulted in a value of 0.625 (Table 3). 

## 4. Discussion

To the best of our knowledge, this is the first multicentric study describing the main factors associated with the prognosis and survival of an equine population submitted to emergency exploratory laparotomy in Spain.

One relevant result in this study was the survival rate of 63.6% in the short term, referring to the time at which the horse was discharged from the hospital. A similar finding has been reported in similar studies, which provide an overall survival rate at discharge between 48.0% and 85.0% [16,17,18,19,20,21]. The proportion of horses that were euthanized or died during the surgical procedure was 22.3%, which is also concurrent with similar studies [14]. 

Post-operative complications have been widely studied [17,22], as their presence or absence is very closely associated with the survival at discharge of any equine patient. We were unable to carry out this analysis due to the multicentric, retrospective nature of our study and, therefore, the lack of data, as well as the lack of consensus regarding the definition of certain complications, such as post-operative reflux or incision infection [16,23,24,25,26,27].

For the multivariable pre-operative model, the following variables were selected: season, patient age, and geographic origin. Based on this pre-operative model, we can assert that the horses surgically treated in summer and winter had a higher risk of death than those operated on in the milder seasons (i.e., spring and fall). Patients aged 9 years or older had a higher risk of death than younger horses, and patients coming from a distance of over 70 km from the surgical referral center had a higher risk than local patients. Age has been shown to be associated with a higher probability of death [21,28], although it has been argued that geriatric patients have the same probability of survival as mature horses [29]. The season has also been found to be associated with a higher probability of post-surgical complications [5,14,17]. However, we found no other studies that concluded, as we did, that either season (summer or winter) was associated with a high risk of death. Further studies are required to assess this finding in other geographic locations. The variable “distance to surgical center”, which was converted into “same region as the surgical referral center or different region”, was used as a proxy for the length of time required to transport the horses. This variable has limitations, as many factors can influence the transport time (e.g., type of vehicle, availability of a trailer, type of road). Still, barn proximity to hospital has been previously reported as a factor that positively influences survival [30,31], which was also confirmed in our study. 

For the multivariable intra-operative model, two new variables (with respect to the pre-operative model) were added: the type (strangulating or non-strangulating) and location (small or large intestine) of the lesion. These two variables have been previously found to be associated with an increased probability of death when the lesions are strangulating or are presented in the small intestine [10,14,17,32,33]. We obtained similar findings, showing that strangulating lesions and small intestine lesions are associated with higher possibilities of death during the intra-operative period.

In the multi-variable COX model generated from the survival rates over the hospitalization period, two variables were found to have a higher predictive power. The classification of the lesion as strangulated and the particular center at which the operation took place were selected in the final multivariable model as the variables with most influence over survival during hospitalization. These results are in accordance with other studies. Strangulating lesions in particular have been associated with death [32,33]. Differences between surgeons and centers have been previously reported [34,35]; however, the large number of variables that can differ between two operating facilities during surgery, and even more during hospitalization, make it difficult to establish an objective distinction between centers. The influence of the clinical center was considered a statistical finding, but, given the lack of considerable clinical data, it was difficult to establish the objective and clinical significance of the difference between centers.

Previously studies in which risk factors have been assessed have predominantly used univariate statistical designs [10]. To our knowledge, there has only been one study similar to the present study [14], in which a multivariable model considering two equine hospitals was used. The present study makes a significant contribution, as our results can be compared with those of similar articles considering other populations with different characteristics. It should be noted that we expected more significant differences regarding horse breed, due to the high prevalence of Spanish Pure Breed horses in our population, as has been previously established [17]. We assume that the multivariate design allowed us to eliminate confusion factors and to select the factors with the greatest predictive power.

In our study, we selected only simple variables for several reasons. The first was the multicentric and retrospective nature of the study, making it is easier to perform with very simple variables. The second reason was the fact that we were interested in identifying the most accurate method for predicting survival based on the simplest available data, in order to provide a practical technique for the daily clinical daily use when a prognosis is requested by an owner based on limited data. Finally, we believe that this study will be more easily reproducible in one or more centers than larger studies using variables that are less likely to be equally available in different centers and locations; for example, parameters involving a physical examination, anesthesia, or blood test analysis. To our knowledge, two investigations with a similar number of horses have been carried in Spain [17,18], but they differed from our study in several ways, such as the inclusion of weather-related parameters and more than one referral hospital. 

The possibility that the weather influences colic episodes in certain regions has been widely studied, but an obvious association has not been found, nor has the weather been shown to be a risk factor for survival following a surgical procedure. It would be interesting to examine variables such as the ambient temperature and atmospheric pressure. In Spain, summer and the month of August have been reported as the periods with the highest probability of post-operative complications [17]. Related to this finding, in our study, the more extreme seasons (winter and summer) were associated with a higher number of surgically treated horses. 

The main limitation of this study was its retrospective nature and, consequently, the lack of some additional data. In the future, it is important to record certain variables, such as whether the horse was euthanized or died spontaneously; the reason for the euthanasia decision (economic restrictions, sentimental issue, poor prognosis); and whether the owner had veterinary medical insurance for the horse. Another limitation was the use of the distance from the horse’s barn to the surgical center as a measure of time, as several bias factors can affect this variable (e.g., time to reach transport, type of road, and type of vehicle). Relevant complications, such as the need to perform a re-laparotomy, also need to be considered in further studies.

## 5. Conclusions

In this study, we evaluated the simple key variables affecting a specific population, identifying several general risk factors that can be very useful when communicating with owners or veterinarians regarding prognosis. The multivariable models generated in our study had a good predictive capacity. The main risk factors associated with intra-operative mortality were the more extreme seasons (winter and summer), patient age (older than 9 years), distance from the hospital, the presence of a strangulating lesion, and the bowel segment affected (small intestine). Furthermore, the main factors associated with mortality during hospitalization were the characteristics of the lesions (strangulating) and the differences between surgical centers. These models, based on simple variables related to the patient (i.e., age, type of lesion, and location of lesion) and to the environment (i.e., seasonality and geographic origin), can be of substantial clinical aid when providing a survival prognosis.

## Figures and Tables

**Table 1:** Multivariable logistic model for pre-surgical factors.

Variable	Category	Odds Ratio	Lower 95% Confidence Interval	Upper 95% Confidence Interval	*p-*Value
Pre-Surgical Model (Hosmer–Lemeshow Test *p*-Value = 0.955, AUC = 0.635; 95% CI = 0.578–0.692)					
Season	Spring/fall	1			0.006
	Summer/winter	1.8	1.2	2.8	
Patient’s age	≤9 years old	1			0.001
	>9 years old	2.0	1.3	3.1	
Geographic origin	Hospital’s region	1			0.023
	Other region **	1.7	1.1	2.7	

** Other region: the approximate distance (in km) was calculated, with 70 km as the cut-off point to determine the category.

**Table 2:** Multivariable logistic model for intra-surgical factors.

Variable	Category	Odds Ratio	Lower 95% Confidence Interval	Upper 95% Confidence Interval	*p-*Value
Intra-Surgical Model (Hosmer–Lemeshow Test *p*-Value = 0.901, AUC = 0.756; 95% CI = 0.707–0.805)					
Season	Spring/fall	1			0.017
	Summer/winter	1.8	1.1	2.8	
Patient’s age	≤9 years old	1			0.016
	>9 years old	1.8	1.1	2.8	
Lesion classification	Non-strangulating	1			<0.001
	Strangulating	5.3	2.7	10.0	
Lesion location	Large intestine	1			0.041
	Small intestine	1.7	1.0	3.0	

**Table 3:** Multivariable COX model for hospitalization-associated factors.

Variable	Category	Hazard Ratio	Lower 95% Confidence Interval	Upper 95% Confidence Interval	*p*-Value
Harrel’s C Test = 0.625					
Lesion classification	Non-strangulating	1			0.004
	Strangulating	1.5	0.1	2.4	
Surgical center	Center A	1			<0.001
	Center B	2.6	1.6	4.1	

## Data Availability

Data available on request due to restrictions privacy.
